# Mice Lacking GABA_A_ Receptor *δ* Subunit Have Altered Pharmaco-EEG Responses to Multiple Drugs

**DOI:** 10.3389/fphar.2021.706894

**Published:** 2021-06-21

**Authors:** Milo Grotell, Shamsiiat Abdurakhmanova, Lauri V. Elsilä, Esa R. Korpi

**Affiliations:** ^1^ Department of Pharmacology, Faculty of Medicine, University of Helsinki, Helsinki, Finland; ^2^ Department of Anatomy, Faculty of Medicine, University of Helsinki, Helsinki, Finland

**Keywords:** pharmaco-EEG, extrasynaptic GABAA receptors, ethanol, opioids, stimulants, psychedelics

## Abstract

In the brain, extrasynaptically expressed ionotropic, *δ* subunit-containing γ-aminobutyric acid A-type receptors (δ-GABA_A_Rs) have been implicated in drug effects at both neuronal and behavioral levels. These alterations are supposed to be caused *via* drug-induced modulation of receptor ionophores affecting chloride ion-mediated inhibitory tonic currents. Often, a transgenic mouse model genetically lacking the δ-GABA_A_Rs (δ-KO) has been used to study the roles of δ-GABA_A_Rs in brain functions, because a specific antagonist of the δ-GABA_A_Rs is still lacking. We have previously observed with these δ-KO mice that activation of δ-GABA_A_Rs is needed for morphine-induced conditioning of place preference, and others have suggested that δ-GABA_A_Rs act as targets selectively for low doses of ethanol. Furthermore, activation of these receptors *via* drug-mediated agonism induces a robust increase in the slow-wave frequency bands of electroencephalography (EEG). Here, we tested δ-KO mice (compared to littermate wild-type controls) for the pharmaco-EEG responses of a broad spectrum of pharmacologically different drug classes, including alcohol, opioids, stimulants, and psychedelics. Gaboxadol (THIP), a known superagonist of δ-GABA_A_Rs, was included as the positive control, and as expected, δ-KO mice produced a blunted pharmaco-EEG response to 6 mg/kg THIP. Pharmaco-EEGs showed notable differences between treatments but also differences between δ-KO mice and their wild-type littermates. Interestingly mephedrone (4-MMC, 5 mg/kg), an amphetamine-like stimulant, had reduced effects in the δ-KO mice. The responses to ethanol (1 g/kg), LSD (0.2 mg/kg), and morphine (20 mg/kg) were similar in δ-KO and wild-type mice. Since stimulants are not known to act on δ-GABA_A_Rs, our findings on pharmaco-EEG effects of 4-MMC suggest that δ-GABA_A_Rs are involved in the secondary indirect regulation of the brain rhythms after 4-MMC.

## Introduction

Pharmaco-electroencephalography (pharmaco-EEG) has been used as a method for distinguishing compounds between different drug classes in early drug development by observing changes in neural oscillations (Dimpfel et al., 1986; Dimpfel, 2003; Drinkenburg et al., 2015). The method is based on the principle that modulation of various neurotransmitter systems affects extracellular field potentials differently (Buzsáki et al., 2012). For example, opioid-like drugs generate a wide suppression of low-frequency bands in pharmaco-EEG, most likely indirectly *via* dopaminergic activation (Dimpfel et al., 1989; Ferger and Kuschinsky, 1995). This effect might be produced by disinhibitory mechanism, as opioids are known to presynaptically inhibit the release of the main inhibitory transmitter γ-aminobutyric acid (GABA) (Depaulis et al., 1987; Bajo et al., 2011). GABA appears as an essential regulator of neuronal excitability, especially *via* its fast-acting ionotropic A-type receptors (GABA_A_Rs) (Olsen and Sieghart, 2009).

The GABA_A_Rs mainly mediate inhibition of neuronal activity by increasing the influx of chloride ions through the receptor ionophore leading to hyperpolarization (Korpi et al., 2002a; Olsen and Sieghart, 2009). GABA_A_Rs are located in synapses, perisynaptically and extrasynaptically, depending on the subunit combinations of the pentameric assemblies. Synaptic GABA_A_Rs often contain a γ_2_-subunit and are sensitive to benzodiazepines, while those located extrasynaptically often include a δ-subunit and are benzodiazepine-insensitive (Brickley and Mody, 2012). Both of those receptor types are abundant, e.g., in the cortex and thalamus (Wisden et al., 1992). In pharmaco-EEG, the activation of synaptic GABA_A_Rs by benzodiazepines increases beta frequency power, whereas the activation of extrasynaptic δ-subunit-containing GABA_A_Rs (δ-GABA_A_Rs) with a superagonist gaboxadol [THIP; 4,5,6,7-tetrahydroisoxazolol(4,5-c)pyridine-3-ol] causes a robust delta power increment (van Lier et al., 2004; Winsky-Sommerer et al., 2007). Thus, pharmaco-EEG can be used to distinguish between ligands acting on different types of GABA_A_ receptors. Notably, the δ-subunit-containing receptors have a higher sensitivity to GABA-site agonists, and therefore, those receptors can respond to changes in extracellular ambient GABA levels (around 0.5 μM) that can be a small fraction of the synaptically released GABA of approximately 1 mM (Mody et al., 1994; Zheleznova et al., 2009; Benkherouf et al., 2019). Sensitivity of δ-GABA_A_Rs is further modulated by the other subunits in the pentameric assembly and, for example, *α*_4/6_ *βδ* receptors are more sensitive than *α*_1_ *βδ* (Zheleznova et al., 2009). These properties might make the extrasynaptic δ-GABA_A_Rs sensitive to the effects of a number of drugs, which could be monitored by pharmaco-EEG.

The δ-GABA_A_Rs have been associated with postpartum depression, a state where endogenous neurosteroid levels are reduced (Ragguett et al., 2019). Accordingly, exogenous neurosteroid analogs that preferentially activate δ-GABA_A_Rs (Mihalek et al., 1999) have shown efficacy in treatment of postpartum depression (Ragguett et al., 2019; Azhar and Din, 2021). More generally, a single nucleotide polymorphism in the GABRD gene (a gene encoding δ-GABA_A_R subunit) has been observed to affect responses to antidepressant drugs (Pu et al., 2013), and emerging evidence suggests that δ-GABA_A_Rs are involved in other mental and neurological disorders as well as in induction of drug dependencies (Arslan, 2021). These associations with δ-GABA_A_Rs and neuropsychiatric disorders make it important to study whether δ-GABA_A_Rs affect various drug-induced responses.

Here we have used cortical pharmaco-EEG to compare wild-type littermate mice (δ-WT) with the δ-GABA_A_R knockout mice (δ-KO) in their responses to multiple drug classes including alcohol, opioids, stimulants, and psychedelics, by monitoring the acute effects (0–2 h after administration) and prolonged or aftereffects (2–5 h) after a single dose. With this approach, the differential effect of THIP has been detected as a robust slow-wave pharmaco-EEG activation in the δ-WT mice, which was lacking in δ-KO mice (Winsky-Sommerer et al., 2007). More recently, we tested the hypothesis of concurrent GABA release from histaminergic neurons leading to an increase in ambient GABA level after the blockade of histamine H_3_ autoreceptors (Yu et al., 2015; Scammell et al., 2019) and found that the δ-KO mice were more sensitive to wake-promoting effects of H_3_ antagonists than the δ-WT mice (Abdurakhmanova et al., 2020), indicating that δ-GABA_A_Rs are involved in restricting the excessive brain excitation induced by the histaminergic system.

First, we tested here the effect of a low dose of ethanol (EtOH) on pharmaco-EEG, as it has been proposed to selectively target the δ-GABA_A_Rs (Wallner et al., 2003), although not consistently (Baur et al., 2009). Furthermore, EtOH has been shown to alter the concentration of extrasynaptic GABA (Jia et al., 2008; Carton et al., 2019). Next, we tested the pharmaco-EEG effects of morphine (MO) since we have recently found that the δ-KO mice are less sensitive to opioid-induced place preference conditioning than the δ-WT mice (Siivonen et al., 2018). We also used a psychoactive, amphetamine-like substituted cathinone mephedrone (4-MMC) (Winstock et al., 2011; Papaseit et al., 2020), which induces similar place preference conditioning in both mouse lines (Siivonen et al., 2018). Finally, we tested the prototype monoamine psychedelic lysergic acid diethylamide (LSD), a known 5-HT_2A_ agonist (González-Maeso et al., 2007), as 5-HT_2A_ agonism is believed to control, at least partially, extrasynaptic GABA concentrations (Abi-Saab et al., 1999). We finished the set of experiments by confirming the robust effect of THIP on pharmaco-EEG in δ-WT mice and the lack of the effect in δ-KO mice.

## Materials and Methods

### Animals

Male δ-KO mice (Mihalek et al., 1999), genotyped and bred in-house, were compared with their wild-type littermates (*n* = 8 and 9, respectively). Only male mice were used to reduce the possibility of producing confounding factors, as the ovarian cycle in female mice regulates the endogenous levels of neurosteroids and the expression of δ-GABA_A_Rs (Bäckström et al., 2003; Maguire et al., 2005). The mice were 13–20 weeks old at the beginning of the experiments. The handling of the animals and the experiments were performed according to national and international guidelines. All experimental procedures were approved by the Animal Experiment Committee of the State Provincial Office of Southern Finland. The mice were housed in single open-air cages with wood chip bedding. Access to food and water was assured *ad libitum*. The health condition of each mouse was evaluated on a daily basis. The EEG recording chambers were in a 12 h:12 h light-dark-cycle (lights on from 06:00 am to 6:00 pm).

### Experimental Drugs

Ethanol (EtOH; 99.99%) and lysergic acid diethylamide (LSD) were acquired from Sigma-Aldrich (St. Louis, MO, United States); morphine (MO) from the University Pharmacy of Helsinki (Helsinki, Finland), and gaboxadol [4,5,6,7-tetrahydroisoxazolo(5,4-c)pyridin-3-ol hydrochloride, THIP] from H. Lundbeck A/S. Mephedrone (4-methyl methcathinone, 4-MMC) was synthesized in-house as described previously (Siivonen et al., 2018). Other compounds, if not otherwise specified, were acquired from Sigma-Aldrich. All drugs were dissolved in physiological saline (0.9% NaCl) and administered intraperitoneally. Solutions were prepared one day prior to the injections.

### Dose Selection

**FIGURE 1:**
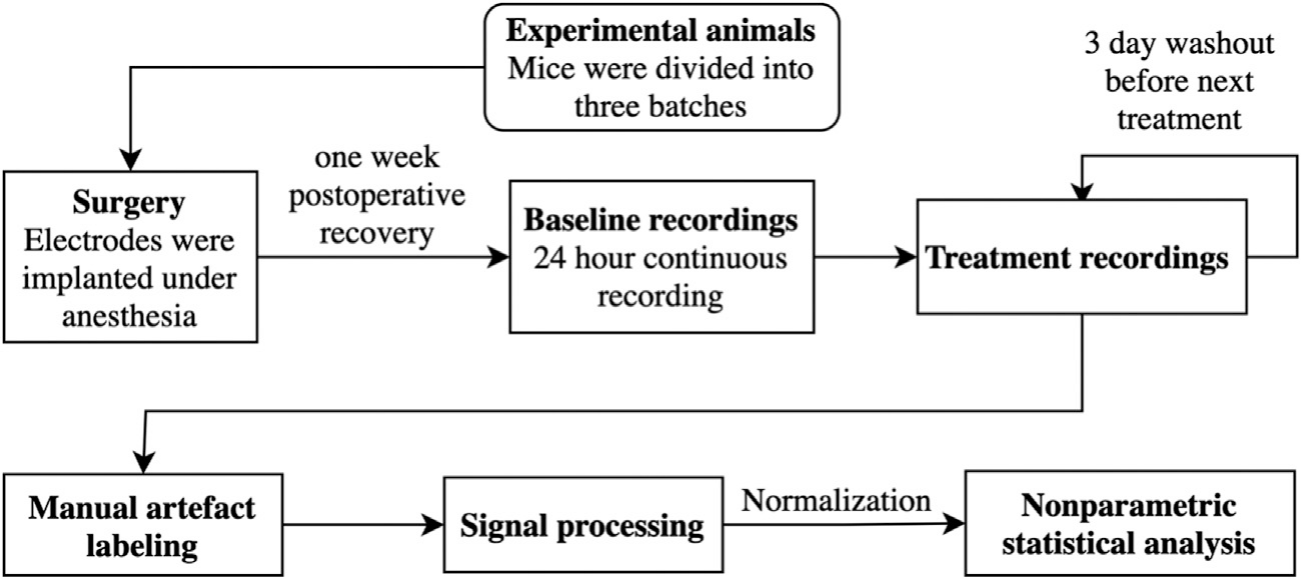
The experimental design is shown as a flowchart.

A low-moderate EtOH dose of 1 g/kg was chosen, as it has been shown to produce blood EtOH concentration of about 0.05–0.1% (by vol, 0.05–0.1 g/dl, 11–22 mmol/L), a range suggested to be relevant for selective activation of δ-GABA_A_Rs (Wallner et al., 2006; Olsen et al., 2007). In addition, the selected dose has been shown to increase brain GABA concentrations, in contrast to higher doses (Carton et al., 2019). A MO dose of 20 mg/kg was chosen because we have previously shown that it produces conditioned place preference (CPP) in WT mice, but not in δ-KO mice, although having enhanced analgetic effects in the δ-KO mice (Siivonen et al., 2018). A 5 mg/kg dose for 4-MMC was chosen as this dose is considered low (Ciudad-Roberts et al., 2015), but still high enough to produce CPP in mice (Siivonen et al., 2018). The dose for the psychedelic LSD was 0.2 mg/kg, which consistently induces robust head-twitch responses as a surrogate to psychedelic drug action (Halberstadt and Geyer, 2013; Halberstadt et al., 2020). A THIP dose of 6 mg/kg was chosen since it has been used to demonstrate that δ-GABA_A_Rs mediate its effects on pharmaco-EEG responses in δ-WT mice but not in δ-KO mice (Winsky-Sommerer et al., 2007). This dose is also active in producing conditioned place aversion in mice (Vashchinkina et al., 2012). The treatments were given in the following order with three-day washout periods: EtOH, Saline (SAL), MO, 4-MMC, LSD, and THIP. The three-day washouts were determined to be long enough as the half-lives of used molecules are maximally a couple of hours. However, as there is evidence that some used compounds like THIP produce long-lasting effects in some brain regions (e.g., the ventral tegmental area) (Vashchinkina et al., 2012), pretreatment baselines used for normalization were analyzed, but no statistical significant differences were detected (χ2, *n* = 93 df = 5, *p: δ*: 5.7E^−01^, *θ*: 5.2E^−01^, *α*: 3.6E^−01^, *σ*: 2.2E^−01^, *β*: 2.1E^−01^, *γ*_1_: 8.5E^−01^, *γ*_2_: 3.1E^−01^). Thus, while we cannot entirely exclude persistent drug-induced alterations in the pharmaco-EEGs with our recording schedule, we could not find any support for this idea by examining the repeated pre-treatment baselines. The experimental design is shown as a flowchart in Figure 1.

### Surgery

EEGs were acquired using intracranial electrodes, which were surgically implanted (Rozov et al., 2016). All surgical procedures were done under isoflurane anesthesia (5% for induction and 1.5–2.5% for maintenance; Attane, Piramal Healthcare, Bethlehem, PA, United States). A dose of an antinociceptive drug (carprofen 5 mg/kg; Rimadyl, Pfizer, United States) was injected 10 min prior to the surgery. First, fur was shaved from the incision location, lidocaine (2%, Orion Pharma, Helsinki, Finland) was injected under the skin, and a midline skin cut was performed on the skull. After incision, *calvaria* was exposed and disinfected using 0.3% hydrogen peroxide. Holes were drilled into the skull bone, and two epidural electrodes were implanted with one electrode placed in the frontal cortex (1.8 mm anterior and 1.3 mm lateral to bregma; *l. dx.*) and the other in the contralateral parietal cortex (1.0 mm anterior and 1.8 mm lateral to lambda; *l. sin.*) for EEG. Two-wire electrodes were implanted into the neck musculature bilaterally for electromyography (EMG). To enhance the adhesion of the dental cement to calvaria, the skull bone was coated with superglue, and the electrodes and screws were secured to the skull with dental cement (Candulor, Wangen, Germany). Postoperative nociception was relieved using buprenorphine (0.1 mg/kg; Temgesic, Reckitt Benckiser, Slough, United Kingdom), injected intraperitoneally.

### EEG Acquisition

After a one-week postoperative period, full-day (24 h) EEG/EMG recordings were acquired using a preamplifier (10,000X gain for EEG) and sampled at 1,000 Hz with counterbalanced cables attached to the previously implanted electrodes. The acquisition of full-day EEG started between 8:00 and 10:00 am. After the full-day baseline recordings, drug treatments were given with three-day washouts. Treatment recordings consisted of 1-h pretreatment EEG and 5-h post-treatment pharmaco-EEG. The acquisition of pretreatment EEGs started between 9:00 and 10:00 am (Zeitgeber time 3–4 h). That time point for pretreatment EEGs was chosen as it has been previously observed that slow-wave NREM has dissipated considerably from the start of the light period (Huber et al., 2000; Wimmer et al., 2013) and that δ-KO and littermate controls have similar patterns of vigilance states (Winsky-Sommerer et al., 2007), making it possible to detect both increases and decreases in slow-wave EEG. For the injections, each animal was individually taken out from its home cage for the injection, and after a quick i.p. drug administration, the animal was placed back into its cage. The same recording settings were used as in the full-day baseline recordings.

### EEG Analysis

EEGs and EMGs were converted into MATLAB file format and exported with Spike2 software (version 8.07, Cambridge Electronic Devices, Cambridge, United Kingdom). The exported files were then imported into MATLAB (MATLAB, 2019) and down-sampled to 200 Hz. The down-sampled files were then manually scored for artifacts using AccuSleep (version November 08, 2020) (Barger et al., 2019). During the manual artifact scoring, the EMGs were utilized alongside the EEGs to determine whether an epoch was affected by an artifact or not.

Signal power analyses were done using the same pipeline structure used in our previous publication (Abdurakhmanova et al., 2020). However, the MATLAB scripts were reimplemented using Python (Van Rossum and Drake, 2009). In short, the scored files were imported into Python using NumPy (Harris et al., 2020). These files were then bandpass-filtered using SciPy Fir-filters (Virtanen et al., 2021). The filters’ windows were constructed as follows: low cutoffs from 1 to 97.6 Hz with 1.4-Hz intervals and high cutoff frequencies from 2.6 to 99.2 Hz with 1.4-Hz intervals. The filtered data were then Hilbert transformed to extract frequency and power information from EEG signal, and median power values were calculated for each 4-s epochs using Pandas (Reback et al., 2020). At this step, the 4-s epochs which contained artifacts were removed.

For the baseline recordings, different frequency bands were averaged over the following frequencies 1–4 Hz for delta (*δ*), 4–8 Hz for theta (*θ*), 8–12 Hz for alpha (*α*), 10–15 Hz for sigma (*σ*), 12–30 Hz for beta (*β*), 30–50 Hz for gamma1 (*γ*_1_) and 50–100 Hz for gamma2 (*γ*_2_). These frequency bins were then averaged to 1-h time bins. These averaged time bins were normalized to the total power over all bands in given 1-h time bins. The averaging and normalization were conducted using Pandas.

For the treatment pharmaco-EEGs, the same band frequencies were used as with the baseline EEGs, but each band was normalized to the average power of pretreatment EEG of the corresponding frequency band.

### Statistics

R (R Core Team, 2020) package NparLD’s (Noguchi et al., 2012) *F2.LD.F1* design was used to analyze general effects of the treatments and *F1.LD.F1* design to analyze effects of the treatments, baselines, or timepoints. Post-hoc analyses were corrected using Bonferroni correction with Python’s statsmodels package (Seabold et al., 2017). Statistical significance was thresholded at *p* < 0.05. Line plots and comparison matrixes were constructed using Seaborn (Waskom et al., 2020).

## Results

### Baseline EEG Spectral Power Analysis

**FIGURE 2:**
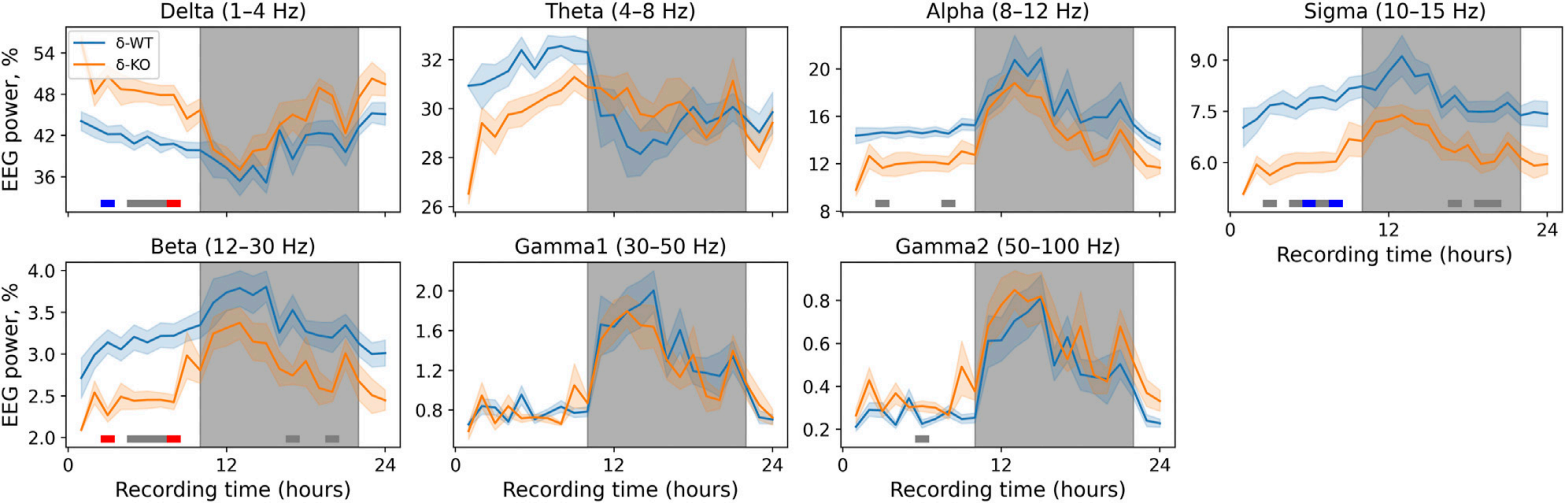
24-h baseline recordings are shown in 1-h time bins. Data are presented as mean ± SEM. Gray underlays show the lights-off period. *p*-values were corrected using the Bonferroni correction for each band individually. The horizontal lines depict statistical significance (*p* < 0.05: gray; *p* < 0.001: blue; *p* < 0.0001: red).

The power of delta and gamma2 bands was increased in δ-KO mice, whereas alpha, sigma, and beta bands were suppressed compared to their littermate WT mice. There were no statistically significant differences in gamma1 and theta bands between the genotypes (χ2, *n* = 17, df = 1, *p: δ*: 3.1E^−03^, *θ*: 2.4E^−01^, *α*: 3.3E^−03^, *σ*: 3.5E^−04^, *β*: 3.2E^−03^, *γ*_1_: 5.7E^−01^, and *γ*_2_: 4.8E^−02^). NparLD failed to produce output for ‘Genotype × Time interaction’ due to a few missing time points caused by a loose connector. Later analyses were, however, done and corrected with conservative Bonferroni correction to minimize the probability for false positives. These corrected statistics of individual time points showed that the alterations were most profound during the light-on period for delta, alpha, and gamma2 bands. Sigma and beta bands were statistically significantly different during light-on and light-off periods. The exact statistically significant time bins are shown in Figure 2.

### Pharmaco-EEG Spectral Power Analysis

**FIGURE 3:**
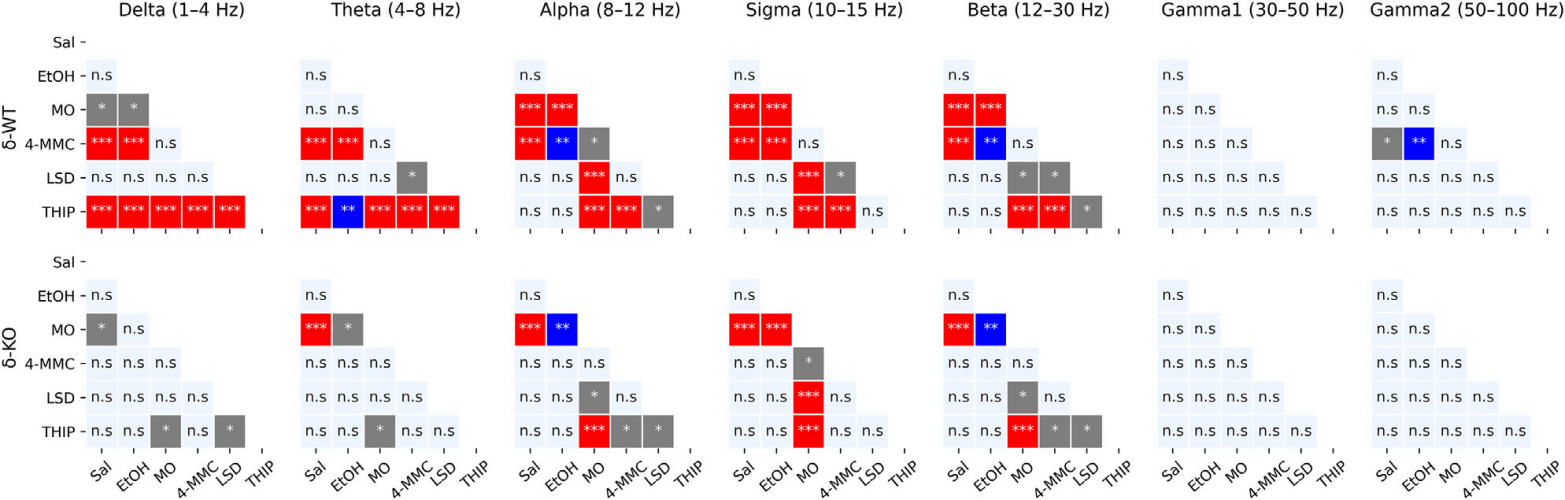
Acute effects (0–2 h) of treatments compared to each other. *p*-values calculated using the Bonferroni correction for each band frequency individually. * < 0.05; ** < 0.001; *** < 0.0001. SAL, saline; EtOH, ethanol; MO, morphine; 4-MMC, mephedrone; LSD, lysergic acid diethylamide; and THIP, gaboxadol.

**FIGURE 4:**
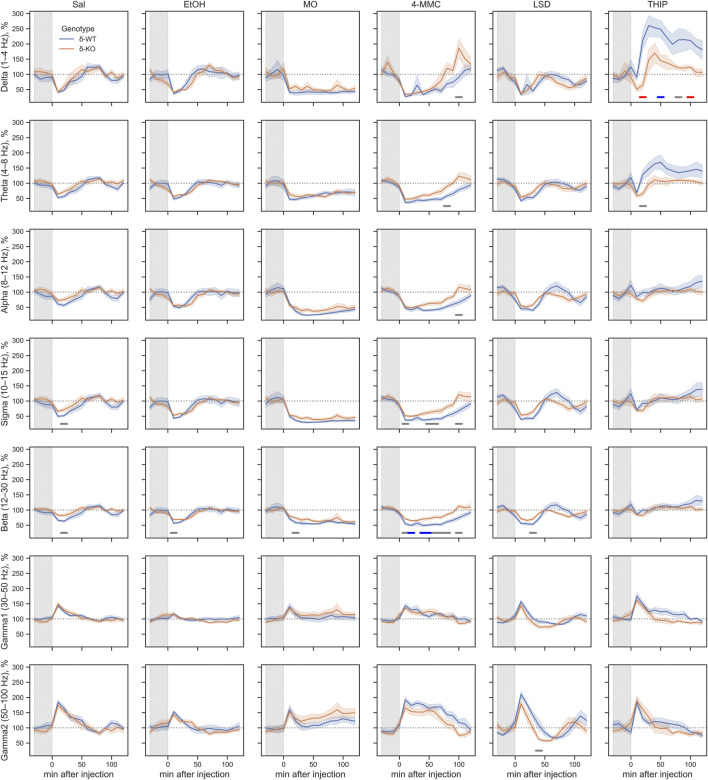
Acute effects of treatments compared between the genotypes during 0–2 h after injection. The gray overlay shows the pretreatment recording period. Plots show mean ± SEM. All treatments are normalized to pre-injection mean individually for each mouse. *p*-values were calculated using the Bonferroni correction for each band frequency. The horizontal lines under the time point show statistical significance between genotypes (*p* < 0.05: gray; *p* < 0.001: blue; *p* < 0.0001: red). SAL, saline; EtOH, ethanol; MO, morphine; 4-MMC, mephedrone; LSD, lysergic acid diethylamide; and THIP, gaboxadol.

Generally for all drug treatments, all frequency bands had a statistically significant ‘treatment effect’ during acute phase of 0–2 h (χ2, *n* = 93, df = 5, *p*: *δ*: 1.5E^−37^, *θ*: 1.2E^−21^, *α*: 8.6E^−34^, *σ*: 8.5E^−53^, *β*: 9.1E^−37^, *γ*_1_: 2.1E^−03^, and *γ*_2_: 1.8E^−05^, Figure 3). However, ‘Treatment × Genotype interaction’ was observed only in delta, theta, and beta bands (χ2, *n* = 93, df = 5, *p: δ*: 2.2E^−06^, *θ*: 2.7E^−03^, and *β*: 2.5E^−02^). Furthermore ‘Treatment x Genotype × Time interaction’ was seen in all frequency bands (χ2, *n* = 93, df = 55, *p*: *δ*: 1.8E^−21^, *θ*: 2.9E^−18^, *α*: 5.8E^−15^, *σ*: 7.6E^−25^, *β*: 5.7E^−34^, *γ*_1_: 5.3E^−112^, and *γ*_2_: 2.0E^−44^, Figure 4). These findings confirm that pharmaco-EEG is able to distinct changes between different treatments.

SAL injections were associated, in both genotypes, with pharmaco-EEG power suppression across the delta, theta, alpha, sigma, and beta frequency bands and with overall increment in gamma1 and gamma2 bands (Figure 4). The peak of these alterations was observed in the first 10-min time bin, with the alterations being gradually normalized to the pre-injection baseline. This observed effect is most likely caused by injection and handling stress.

A high dose of EtOH is observed to induce altered behavioral responses in *δ*-KO mice (Mihalek et al., 2001) and has been able to produce changes in cortical field potentials (Abrahao et al., 2020). However, the ‘Treatment effect’ post-hoc analysis showed no signal power alterations induced by a low dose (1 g/kg) of EtOH in δ-WT or δ-KO mice during 0–2 h after administration when compared to SAL (Figure 3). Further analysis regarding ‘Genotype × Treatment interaction’ showed no statistically significant differences between the genotypes. Only one statistically significant time point was observed in the ‘Genotype × Treatment × Time interaction’ post-hoc analysis (Figure 4).

‘Treatment effect’ post-hoc analysis showed MO-induced signal power suppression in delta, alpha, sigma, and beta frequencies during 0–2 h in δ-WT mice, whereas in δ-KO mice, the MO-induced suppressions were also detected in theta band when compared to SAL (Figure 3, ‘SAL vs. MO’; δ-WT (χ2, *n* = 15, df = 1, *pc*: *δ*: 1.1E^−03^, *θ*: 8.0E^−02^, *α*: 7.4E^−18^, *σ*: 3.1E^−16^, and *β*: 3.7E^−07^; δ-KO: (χ2, *n* = 14, df = 1, *pc*: *δ*: 2.6E^−02^, *θ*: 4.2E^−05^, *α*: 7.6E^−04^, *σ*: 1.4E^−12^, and *β*: 3.4E^−09^). ‘Genotype × Treatment interaction’ post-hoc analysis showed no statistically significant effects between the genotypes. These findings are in line with previous pharmaco-EEG studies where MO-induced alterations in field potentials were thought to be mediated *via* indirect DA-receptor activation (Dimpfel et al., 1989; Ferger and Kuschinsky, 1995).

Stimulants have similar locomotor activating, sensitizing, and rewarding effects in δ-WT or δ-KO mice (Siivonen et al., 2018) and, therefore, we expected to see no significant differences in pharmaco-EEG responses after 4-MMC. However, ‘Treatment effect’ post-hoc analysis showed 4-MMC-induced signal power suppression in all band frequencies except gamma1 in δ-WT mice during 0–2 h, whereas 4-MMC-induced suppressions where not observed in δ-KO when compared to SAL (Figure 3, ‘SAL vs. 4-MMC; δ-WT: χ2, *n* = 18, df = 1, *pc*: *δ*: 2.9E^−06^, *θ*: 2.5E^−05^, *α*: 5.0E^−05^, *σ*: 8.6E^−06^, *β*: 1.8E^−05^, *γ*_1_: 1.0E^+00^, and *γ*_2_: 3.5E^−02^; δ-KO: χ2, *n* = 14, df = 1, *pc*: *δ*: 1.0E^+00^, *θ*: 3.8E^−01^, *α*: 5.0E^−02^, *σ*: 2.1E^−01^, *β*: 8.9E^−02^, *γ*_1_: 1.0E^+00^, and *γ*_2_: 1.0E^+00^). ‘Genotype × Treatment interaction’ post-hoc analysis showed statistically significant effects between the genotypes in theta and beta bands (Genotype × Treatment interaction: χ2, *n* = 17, df = 1, *pc*; *θ*: 2.1E^−02^, and *β*: 2.5E^−04^). Lastly, ‘Genotype × Treatment × Time interaction’ post-hoc analysis showed notable differences in sigma and beta bands (Figure 4).

Albeit activation of 5-HT_2_ receptors have been shown to alter extrasynaptic GABA levels (Abi-Saab et al., 1999), ‘Treatment effect’ post-hoc analysis showed no LSD-induced signal power alterations during 0–2 h in δ-WT or δ-KO mice when compared to SAL (Figure 3). Not surprisingly, ‘Genotype × Treatment interaction’ post-hoc analysis showed no statistically significant differences between the genotypes. One statistically significant time point was observed in ‘Genotype × Treatment × Time interaction’ post-hoc analysis as shown in Figure 4.

To confirm that the pharmaco-EEG of the experimental batch of mice responded as shown previously (Winsky-Sommerer et al., 2007), we administered THIP at the dose of 6 mg/kg to provoke substantive increases in the delta band EEG in the wild-type mice with little effect on that of the δ-KO mice. ‘Treatment effect’ post-hoc analysis showed THIP-induced signal power increment in delta and theta frequency bands during 0–2 h in δ-WT mice, whereas in δ-KO mice, no THIP-induced alterations were detected compared to SAL (Figure 3, ‘SAL vs. THIP’; δ-WT: χ2, *n* = 17, df = 1, *pc: δ*: 4.0E^−22^ and *θ*: 2.7E^−05^; *δ*-KO: χ2, *N* = 15 df = 1, *pc: δ*: 1.0E^+00^ and *θ*: 1.0E^+00^). Also, ‘Genotype × Treatment interaction’ post-hoc analysis showed that δ-WT was statistically different from δ-KO (χ2, *n* = 15, df = 1, *pc: δ*: 7.2E^−07^ and *θ*: 2.6E^−02^). Lastly, ‘Genotype × Treatment × Time interaction’ post-hoc analysis is shown in Figure 4. These results indicate that the mouse model that we used responded to the δ-GABA_A_R superagonist THIP as predicted.

**FIGURE 5:**
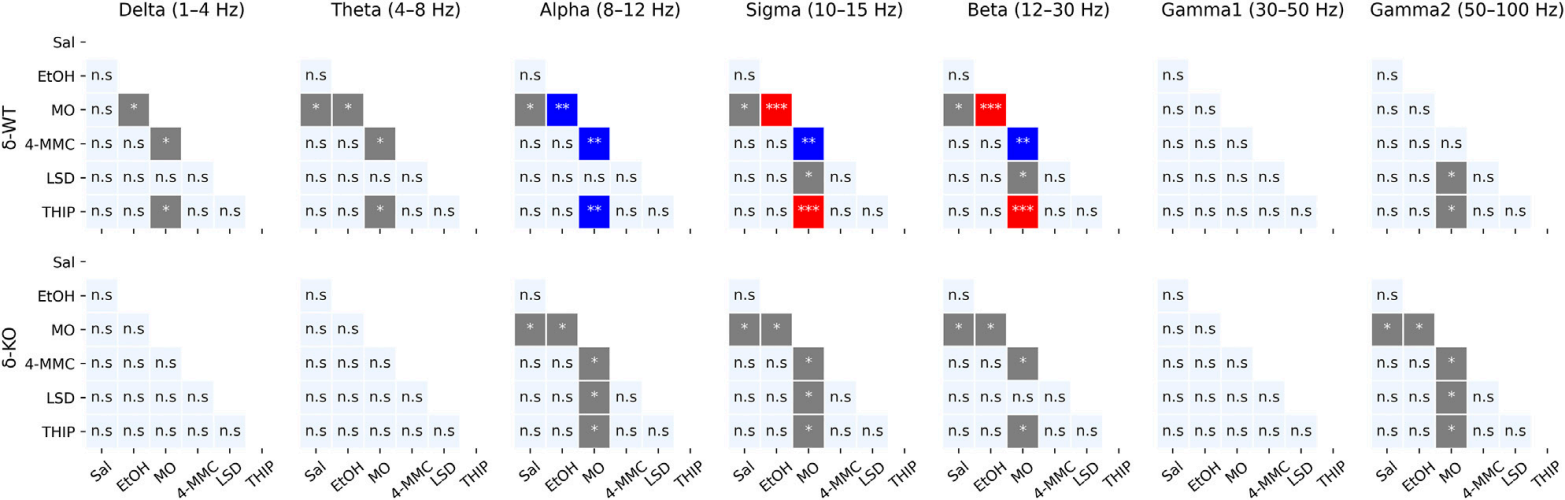
Sub-acute effects (2–5 h) of treatments compared to each other. *p*-values were calculated using the Bonferroni correction for each band frequency individually. * < 0.05; ** < 0.001; *** < 0.0001. SAL, saline; EtOH, ethanol; MO, morphine; 4-MMC, mephedrone; LSD, lysergic acid diethylamide; and THIP, gaboxadol.

**FIGURE 6:**
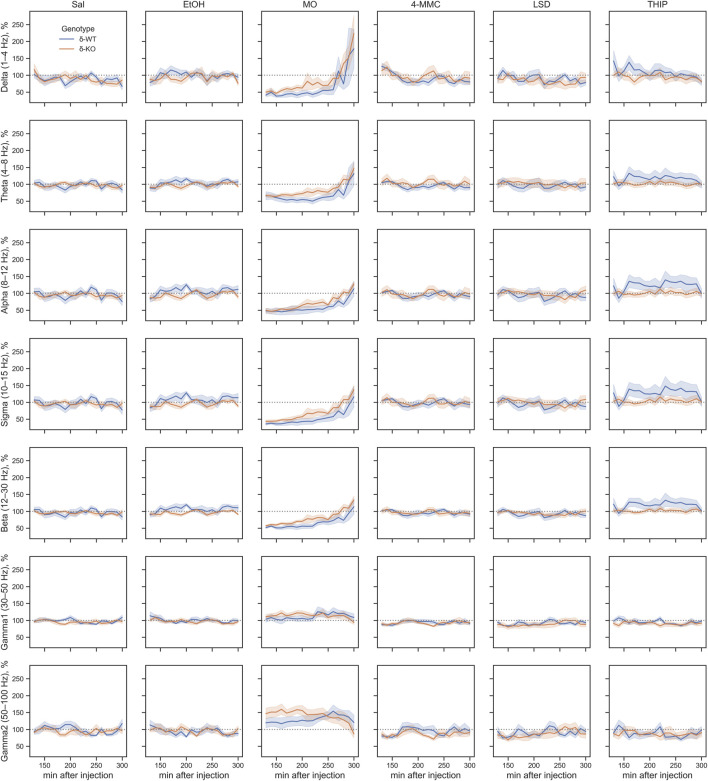
Sub-acute effects (2–5 h after injection) of treatments compared between genotypes. Plots are showing mean ± SEM. All treatments are normalized to pre-injection mean individually for each mouse. SAL, saline; EtOH, ethanol; MO, morphine; 4-MMC, mephedrone; LSD, lysergic acid diethylamide; and THIP, gaboxadol. No differences were observed between genotypes due to the lack of the main effect.

In the following subacute observation period (2–5 h) all frequency band-wide ‘Treatment effects’ remained (χ2, *n* = 93, df = 5, *p: δ*: 1.8E^−04^, *θ*: 1.7E^−05^, *α*: 1.2E^−09^, *σ*: 2.4E^−11^, *β*: 2.1E^−12^, *γ*_1_: 6.4E^−04^ and *γ*_2_: 4.0E^−07^). However, only the effect of MO treatment remained statistically significant from that of SAL in both genotypes (Figures 5, 6).

## Discussion

Here we observed that δ-KO mice have notable differences in drug-induced pharmaco-EEG responses. These findings were not limited to the previously shown blunted response to THIP (Winsky-Sommerer et al., 2007), but the δ-KO mice were also less sensitive to 4-MMC-induced suppression of pharmaco-EEG across multiple frequency bands. Furthermore, we observed similarly altered baseline EEG band powers as in our previous experiments with the same δ-KO mouse line (Abdurakhmanova et al., 2020), indicating that cortical EEG is a well reproducible method to study drug-induced alterations in cortical field potentials.

Morphine (MO) at the dose of 20 mg/kg induced suppression in multiple low-frequency bands, a finding that is in line with previous research. It has been suggested that these alterations are caused by indirect activation of dopamine (DA) receptors, but they can also be dramatically attenuated with non-selective opioid receptor antagonists (Dimpfel et al., 1989; Ferger and Kuschinsky, 1995). More interestingly, a rebound of mainly delta band activity was observed similarly in both genotypes at the end of prolonged MO effects (2–5 h). Previous research has observed an increase in delta frequency band induced by nitrous oxide and ketamine, known N-methyl-D-aspartate (NMDA) receptor antagonists, and this pharmaco-EEG-effect has been associated with the rapid antidepressant effect of these drugs (Kohtala et al., 2019). A similar increment in slow-wave EEG is also observed after electric therapies for depression (Sackeim et al., 1996). Current knowledge suggests that many unconventional fast-acting antidepressants, working *via* NMDA receptor antagonism, rely on their interactions with the opioid system and that non-selective antagonism of the opioid receptors abolishes drug-induced antidepressant effects (Ragguett et al., 2019; Dolatshahi et al., 2020; Klein et al., 2020; Perez-Caballero et al., 2020). Kohtala et al. (2019) have, however, suggested that the rapid antidepressant effects require, in addition to slow-wave EEG increment, upregulation of TrkB and GSK3β signaling pathways. All this combined has raised an important question of whether known opioid system agonists like MO could be used to treat depression. Indeed, some previous research has shown that subclinically depressed people have a lower number of μ-opioid receptors and that opioid agonists reduce depression, although with a cost of liability to addiction and tolerance, which has strongly limited their use (Tejedor-Real et al., 1995; Ragguett et al., 2019; Nummenmaa et al., 2020; Perez-Caballero et al., 2020). In spite of these problems, an opioid agonist/antagonist combination (buprenorphine + samidorphan) has been developed to treat treatment-resistant depression and is currently in clinical trials (Ragguett et al., 2018; Ragguett et al., 2019). Also, the antidepressant tianeptine, working at least partly *via* μ-opioid receptor agonism, has proven effectiveness in treating depression (Samuels et al., 2017). When taking together with previous knowledge that δ-KO mice show blunted MO-induced conditioned place preference (Siivonen et al., 2018), we can hypothesize that combining opioids with δ-GABA_A_Rs antagonist could provide a novel and a better-tolerated way to make opioids an alternative way to treat depression. Unfortunately, at present, there are no known selective δ-GABA_A_R antagonists.

Alternatively, delta frequency band increment can be induced *via* direct δ-GABA_A_Rs agonism with THIP, as shown here and confirming the results (Winsky-Sommerer et al., 2007). Not surprisingly, we similarly observed a diminished THIP-induced signal power increment in delta and theta frequency bands in δ-KO mice. A study by Maguire and Mody (Maguire and Mody, 2008) demonstrated that reduced activation of δ-GABA_A_Rs has a role in postpartum depression, observing that δ-KO mice have increased depression-like behaviors and an implicit failure in normal maternal behaviors. Brexanolone (allopregnanolone), a neurosteroid agonist which preferentially activates δ-GABA_A_Rs (Mihalek et al., 1999), has shown efficacy in the treatment of postpartum depression, a condition where endogenous neurosteroids are downregulated in humans (Ragguett et al., 2019; Arslan, 2021; Azhar and Din, 2021). Rising evidence also suggests that brexanolone could be an effective treatment for other types of mood disorders as well (Arslan, 2021).

Although it has been shown that δ-KO mice have altered behavioral responses to high doses of EtOH (Mihalek et al., 2001), others have suggested δ-GABA_A_Rs to be selective targets for low EtOH doses/concentrations (Wallner et al., 2003; Wallner et al., 2006; Olsen et al., 2007). It is also known that EEG can detect EtOH-induced changes in local field potentials (Abrahao et al., 2020). Therefore, it was, essential to study the actions of low EtOH doses on the pharmaco-EEG in this model. Unfortunately, both genotypes showed similar pharmaco-EEG responses to 1 g/kg of EtOH, which is surprising because a low dose of EtOH has been shown to alter the concentration of extrasynaptic GABA (Jia et al., 2008; Carton et al., 2019). However, EtOH treatment did not differ from saline control treatment either. That suggests that cortical pharmaco-EEG is not sensitive enough to detect small changes in GABA concentration induced by EtOH or that EtOH-induced alterations mostly happen in subcortical brain areas not detectable by cortical pharmaco-EEG. On the other hand, EtOH effects on δ-GABA_A_Rs have been challenging to reproduce in various models (Borghese et al., 2006; Korpi et al., 2007), making it less likely that low alcohol doses would have selective effects on this receptor subtype. Since it is known in mouse and rat models that sleep is promoted both by acute moderate to high doses of EtOH or binge drinking and impaired by EtOH withdrawal (Thakkar et al., 2015), further studies are needed to assess the effects of low doses of EtOH on sleep EEG and the role of δ-GABA_A_Rs on effects of higher EtOH doses in our mouse model.

Although stimulants have in the brain other main targets than GABA_A_Rs, such as monoamine transporters (Korpi et al., 2015), the neuronal circuitries they affect often constitute GABAergic neurons and pathways like medium spiny neuron populations in the striatum. Furthermore, e.g., cocaine reward and sensitization are associated with α_2_-GABA_A_R responses (Dixon et al., 2010) and methamphetamine self-administration is enhanced by benzodiazepines acting on GABA_A_Rs (Spence et al., 2016). The roles of the δ-GABA_A_Rs in stimulant actions have been little studied before. Here, we also observed that pharmaco-EEG of δ-WT mice after treatment with 5 mg/kg of 4-MMC was suppressed at multiple low-frequency bands and gamma2 band, which was not seen in δ-KO mice. Interestingly, the most notable changes were observed as beta frequency band suppression, as an increased power of beta band has previously been associated with the increased synaptic GABA_A_R activation by benzodiazepines (van Lier et al., 2004). The lack of observed suppression in δ-KO mice could thus be due to decreased sensitivity to changes in extrasynaptic GABA concentrations compared to their wild-type littermates. Alternatively, an increased number of *γ*_2_ subunit-containing synaptic GABA_A_Rs (γ_2_-GABA_A_Rs) due to adaptations to loss of δ-subunit in δ-KO mice could make δ-KO mice more sensitive to changes in synaptic GABA levels (Korpi et al., 2002b; Peng et al., 2002). When considering that MO-induced pharmaco-EEG alterations are supposed to be mediated *via* indirect DA effect, we hypothesized that the 4-MMC-induced alterations are, in addition to the direct DA effect, affected by GABAergic inhibitory feedback mechanisms (Dimpfel et al., 1989; Ferger and Kuschinsky, 1995). On the other hand, δ-KO mice show normal sensitivity to locomotor activation as well as induction and expression of conditioned place preference to stimulants methamphetamine and 4-MMC (Siivonen et al., 2018), suggesting dissociation of the altered pharmaco-EEG response from the psychomotor stimulation and rewarding effects.

Even though extrasynaptic GABA concentrations have been shown to be at least partially controlled by the activation of the 5-HT_2A_ receptors (Abi-Saab et al., 1999), no pharmaco-EEG differences between the genotypes were observed when mice were treated with the prototype serotonergic psychedelic LSD, a known 5-HT_2A_ agonist, with a head-twitch-inducing dose of 0.2 mg/kg (González-Maeso et al., 2007; Halberstadt and Geyer, 2013). In a study by Abi-Saab et al. (1999), extrasynaptic GABA concentrations were increased acutely by a dose-dependent manner with DOI (2,5-dimethoxy-4-iodoamphetamine), a known 5-HT_2A/C_ agonist, but they normalized very rapidly during the first hour. This fast normalization of extrasynaptic GABA might be the reason why no differences were observed in pharmaco-EEG between genotypes – all possible differences induced by LSD could have been muddled by the alterations caused by possible stress effects of i.p. administration, which was most pronounced during the first hour.

One major limitation of conventional δ-KO mice is the presence of major developmental adaptations to the lack of the GABA_A_R δ-subunit (Korpi et al., 2002b; Peng et al., 2002; Spigelman et al., 2002). These alterations are not limited to the altered full-day baseline EEG activity, which is thought to be acquired due to more hyperpolarized thalamocortical neurons and the previously assessed increment in the synaptic γ_2_-GABA_A_Rs (Mesbah-Oskui et al., 2014). These δ-KO mice also show a significantly reduced response to stress, reduced episodic memory consolidation, and depression-like behaviors as adaptations (Spigelman et al., 2002; Maguire and Mody, 2008; Sarkar et al., 2011; Mesbah-Oskui et al., 2014). δ-KO mice have also reduced expression of *α*_4_ subunits that are often co-expressed in δ-GABA_A_R assemblies (Korpi et al., 2002b). This reduced expression of *α*_4_ subunits could further reduce sensitivity to extrasynaptic ambient GABA, but as the δ-KO mice show also increased level of benzodiazepine-insensitive *α*_4_ *βγ*_2_ receptors, the loss of *α*_4_ subunits is at least partially compensated by its increased receptor incorporation with available γ_2_ subunits (Korpi et al., 2002b; Peng et al., 2002). The main *α* subunit, the *α*1, is increased in the striatum and cerebellum of the δ-KO mice, but unaltered in many other regions (Tretter et al., 2001; Korpi et al., 2002b; Peng et al., 2002). These adaptations could also be behind the altered pharmaco-EEG responses in δ-KO mice, but unfortunately, there are no selective antagonists for GABA_A_R subtypes that could be directly used to evaluate the pharmacological roles of these receptors (Korpi et al., 2002a). Therefore, this limitation should be taken into consideration when interpreting the results.

To sum up, δ-KO mice were less sensitive to THIP-induced alterations in cortical pharmaco-EEG and had blunted responses to 4-MMC-induced power suppression. The exact mechanisms of drug actions on EEG remain uncertain due to the descriptive nature of pharmaco-EEG, with the exception of THIP, which is known to be a superagonist of δ-GABA_A_Rs (Stórustovu and Ebert, 2006; Saarelainen et al., 2008). Furthermore, δ-KO mice had a similar pharmaco-EEG response to EtOH, LSD, and MO as compared to their wild-type littermates, which suggests that cortical pharmaco-EEG in mice is not particularly sensitive to smaller alterations in ambient GABA levels around the δ-GABA_A_Rs. It remains to be studied whether these findings could help to provide novel ways to treat depression and stress as well as reduce drug-caused harm.

## Data Availability

The raw data supporting the conclusions of this article will be made available by the authors, without undue reservation.
